# Study Processes and Applications of Ultrasomics in Precision Medicine

**DOI:** 10.3389/fonc.2020.01736

**Published:** 2020-09-03

**Authors:** Rui Yin, Meng Jiang, Wen-Zhi Lv, Fan Jiang, Jun Li, Bing Hu, Xin-Wu Cui, Christoph F. Dietrich

**Affiliations:** ^1^Department of Ultrasound, Affiliated Renhe Hospital of China Three Gorges University, Yichang, China; ^2^Sino-German Tongji-Caritas Research Center of Ultrasound in Medicine, Department of Medical Ultrasound, Tongji Hospital, Tongji Medical College, Huazhong University of Science and Technology, Wuhan, China; ^3^Department of Artificial Intelligence, Julei Technology, Wuhan, China; ^4^Department of Ultrasound, The Second Affiliated Hospital of Anhui Medical University, Hefei, China; ^5^Department of Ultrasound, The First Affiliated Hospital, School of Medicine, Shihezi University, Shihezi, China; ^6^Department of Internal Medicine, Hirslanden Clinic, Bern, Switzerland

**Keywords:** ultrasomics, diagnosis, tumor, ultrasound, artificial intelligence, computer aided diagnosis

## Abstract

Ultrasomics is the science of transforming digitally encrypted medical ultrasound images that hold information related to tumor pathophysiology into mineable high-dimensional data. Ultrasomics data have the potential to uncover disease characteristics that are not found with the naked eye. The task of ultrasomics is to quantify the state of diseases using distinctive imaging algorithms and thereby provide valuable information for personalized medicine. Ultrasomics is a powerful tool in oncology but can also be applied to other medical problems for which a disease is imaged. To date there is no comprehensive review focusing on ultrasomics. Here, we describe how ultrasomics works and its capability in diagnosing disease in different organs, including breast, liver, and thyroid. Its pitfalls, challenges and opportunities are also discussed.

## Introduction

Ultrasomics is the science of transforming digitally encrypted medical images that hold information related to tumor pathophysiology into mineable high-dimensional data (1, 2). The role of ultrasomics is to quantify the diseases using distinctive imaging algorithms and thereby provide valuable information for personalized medicine (3).

The Precision Medicine Initiative was launched in 2015 and studied the complex biological behaviors of tumors and their interactions. This initiative uses a holistic approach to explain the complexity of biological systems and starts with the recognition that the network that makes up an entire organism is not just the sum of its parts (4). In situations where traditional “one-on-one” diagnosis and treatment are unable to meet medical requirements, a multidisciplinary comprehensive diagnosis method is needed for both doctors and patients. This approach incorporates not only the relatively static genetic code but also the dynamic changes and heterogeneous nature of tumors (5). Radiomics plays a key role in precision medicine. Ultrasomics is a branch of radiomics that extracts vast arrays of quantitative features from ultrasound images and integrates them with the clinical data of patients. It can obtain the texture, shape, intensity, trends and wavelet features of a tumor, distinguish heterogeneity between tumors, and provide a comprehensive quantitative tumor phenotype for doctors (6). The aim of ultrasomics is to obtain the optimal efficacy and safety to ensure maximum quality of life and to avoid excessive and ineffective treatments (7). Ultrasomics does not aim to replace existing clinical decision-making tools but to provide a supplement to current measures by implementing a robust, low-cost, repeatable, and highly effective approach to current clinical practice (8).

## Basic Techniques of Ultrasomics Analysis

**FIGURE 1:**
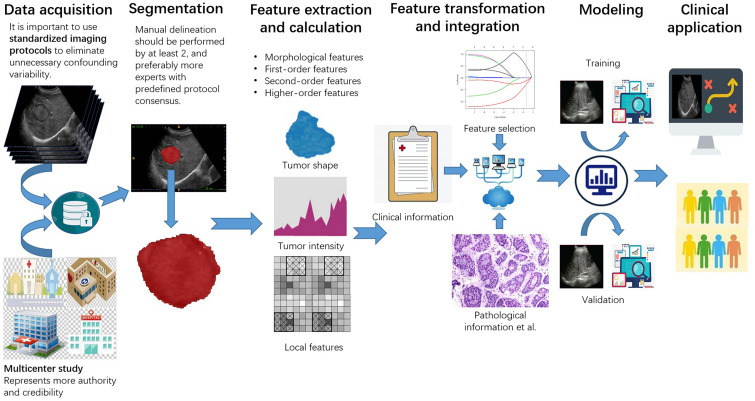
The workflow of ultrasomics. The workflow includes data acquisition, segmentation, feature extraction and calculation, feature transformation and integration, modeling, and clinical application.

Ultrasomics is defined as quantitative mapping, that is, extracting medical imaging features related to predicted targets, analyzing the information contained, and finally establishing a model. The basic steps of ultrasomics include data acquisition, segmentation, feature calculation, and modeling (Figure 1).

### Data Acquisition

Ultrasomics usually begins with a prediction target — the event a doctor wishes to predict. Building a successful model usually relies on access to a large number of medical images and clinical data to reveal correlations. Different data sources may have an unexpected impact on the results, therefore, it is important to use standardized imaging protocols to eliminate unnecessary confounding variability (9).

Data, including images, may be retrospectively collected or prospectively acquired, depending on the study design. The greatest obstacle to the reliability and stability of ultrasomics is the high variability of ultrasound images acquired by different operators. Single-center data are usually best obtained by a few radiologists with just a few machines, which guarantees better image consistency. However, multicenter study represents more authority and credibility as it requires a large number of representative teams to jointly obtain relevant data sets. This requires the participating hospitals to reach a clear agreements and establishment of standardized operating standards, including unifying the machine, frequency of the probe, gain of the image, focus, depth, resolution and gray value, and unifying whether to add blood flow, radiography, and patient posture (10). Retrospective image acquisition currently lacks standardization, and raw data are usually not available. Thus, reconstructed images must be used. Conversely, when images are acquired prospectively, an image acquisition standard suitable for ultrasomics should be selected for analysis. Standards in this situation are controllable and can maximize the information for subsequent work (11).

### Segmentation

Defining the region of interest (ROI) can be undertaken by manual segmentation, semiautomatic segmentation and automatic segmentation (12). Regardless of which method is used, this step is time-consuming and challenging. On the one hand, there is no gold standard for ROI segmentation, and it is difficult to define the morphology, echogenicity and boundary of a variable lesion. On the other hand, a consensus for image standardization is difficult to reach for a diffuse disease or multiple lesions.

Ultrasomics segmentation includes outlining 2-dimensional (2D) ROIs and 3-dimensional (3D) volumes of interest (VOIs). In most studies, experts isolate the object of interest with a manual algorithm. Manual delineation has well-known limitations regarding inter- and intraobserver variability and should be performed by at least 2, and preferably more experts with predefined protocol consensus. This process is undoubtedly tedious, with inevitable variability. In recent years, deep learning has given researchers automatic or semiautomatic segmentation, which outperforms fixed thresholding, aiming to achieve higher accuracy. The common segmentation algorithms include region-growing, level setting, image cutting, active contour (snake) algorithms, semiautomatic segmentation, and livewire methods. However, since automatic segmentation techniques are in the exploratory stage and require much debugging and revision, their applications are still limited. Semiautomatic segmentation is a perfect combination of manual control and intelligence. Region-growing is one of the semiautomatic methods that was often used to the segmentation in computer-aided system (CAD) (13). The method is called “click and grow,” that is, putting the seed points in the target area, then it will automatically grow around and automatically stop at the edge of the lesions. The seed point is generally selected in the center of the target tumor. This segmentation method saves both time and effort, but when the boundary of lesion appears to be unclear, the segmentation results may not be ideal and may need to be modified by a professional radiologist. Regardless of the segmentation method that researchers use, the ultimate aim is based on the reliability of the ROI result.

### Feature Calculation

Ultrasomics features are automatically extracted by computer algorithms from the sketched ROI. The characteristics mined by different research institutes are different and have different content. In general, the features are divided into four parts: morphological features, first-order features, second-order features, and higher-order features.

Morphological features include lesion volume, shape (spherical, non-spherical, etc.), and boundary morphology (flat, round, clear, sharp, fuzzy, amorphous, and unclear, etc.). These features are acquired based purely on the experience of radiologists, but despite this, they still offer many possibilities for generating hypotheses.

The first-order features are the common statistical elements (gray signal-strength value) obtained from the image, which include the average pixel signal value, standard deviation, skewness, and kurtosis. These features are represented by a single value or frequency distribution histogram, which quantitatively summarizes the signal intensity of the target area. Although they have great appeal in ultrasomics due to their simplicity, these features do not include spatial information.

Second-order features are usually described as texture features, which include the gray intensity of adjacent pixels. Second-order features suggest indicative information for cancer judgment and are used to explain the internal heterogeneity and complexity of the spatial distribution of the tumors. Commonly used second-order statistical descriptors include gray-level co-occurrence matrices (GLCMs) and gray-level run length matrices (GLRLMs). In GLCMs, the frequencies adjacent to (co-occurrence) pixels of the same signal strength are provided as a matrix to describe the density of the signal strength in a particular direction to reveal differences in regional heterogeneity (14). In GLRLMs, the heterogeneity of signal strength within the ROI can be determined by calculating the frequency at which the nearest-neighbor pixels match in intensity or the operating frequency with the same signal strength (15).

Higher-order features include filters and higher-order images to describe metrics. These features describe the local spatial organization of signal-strength values by applying and adjusting filters in multidimensional space. This represents a quantitative approach. Higher-order feature elements are usually obtained from gray values by the Fourier transform (FT), which converts spatial information to the frequency space and then reverses the conversion process back to the spatial domain (16). Typical techniques include the discrete orthonormal Stockwell transform (DOST), Gabor filter banks, the wavelet transform (WT), the Riesz transform, the Stockwell transform (ST), and the Laplacian of the Gaussian (17).

### Modeling

Model building for ultrasomics includes three main steps: feature selection, modeling development, and validation. Selecting the required indicators from infinite features and avoiding overfitting should be the main focus. Ultrasomics and non-ultrasomics features should be combined with the prediction target to create a single dataset. This enables the investigation of relationships between features. Feature pruning is usually required, because computing a large number of features from several matrices can result in many redundant and/or highly correlated features, which greatly increases the complexity of the problem without adding useful information (18). Feature extraction is followed by pruning, methods for which usually include (1) the wrapper method, which scores features based on a performance classification to reflect the usefulness of each feature; (2) the filter method, which uses statistical methods to sort the features and select the highest-ranking feature to determine the intrinsic value of each feature; and (3) the embedded method, which is similar to the wrapper method in some aspects because characteristics are selected to optimize the performance of the learning algorithm. However, unlike the wrapper method, which uses the classification method as an external black box to sort the features, variable selection in the embedded method is an inherent part of the learning algorithm itself.

Model development methods are usually based on the skills and experience of the researcher, which has associated limitations. When training the model, the training samples with the corresponding clinical tags are paired with the training model. Through a predefined loss function, the relationships between the learning characteristics of the model and the clinical label are found, and finally, the model with good training results is selected for testing, this is called supervised learning. In unsupervised learning, the training model no longer needs clinical labeling, it divides similar samples into a set of final generated models according to the algorithm.

Verification is a tool to evaluate whether a model is useful. This research is considered successful only when both the internal and external verification results are satisfactory.

With the application of a classifier, it is necessary to use corresponding measures to evaluate the results as a way of verifying the stability of the generated model. Therefore, the measures below are considered for a confusion matrix of true positives (TPs), true negatives (TNs), false positives (FPs), and false negatives (FNs).

The performance of the model is evaluated by the following formulas:

$$S\,e\,n\,s\,i\,t\,i\,v\,i\,t\,y=\frac{T\,P}{T\,P+F\,N}$$

$$S\,p\,e\,c\,i\,f\,i\,c\,i\,t\,y=\frac{T\,N}{T\,N+F\,P}$$

$$A\,c\,c\,u\,r\,a\,c\,y=\frac{T\,P+T\,N}{T\,P+T\,N+F\,P+F\,N}$$

$$F\,1_{-}\,s\,c\,o\,r\,e=2\times p\,r\,e\,c\,i\,s\,i\,o\,n\times \frac{\,S\,e\,n\,s\,i\,t\,i\,v\,i\,t\,y\,}{\,p\,r\,e\,c\,i\,s\,i\,o\,n\,+\,S\,e\,n\,s\,i\,t\,i\,v\,i\,t\,y\,}$$

Additionally, the area under the receiver operating characteristic curve (AUROC) is commonly used to describe the overall performance of a parameter. An AUROC value close to 1 represents an ideal value. A value less than 0.5 suggests that the parameter does not have any classification ability.

## Applications of Ultrasomics in Precision Medicine

The ultimate goal of ultrasomics is to assist radiologists in diagnosing diseases. Currently, studies on ultrasomics cover collecting imaging features, genetic features, and clinical features for data mining analysis and performing tumor screening, diagnosis, classification, and staging predictions. Ultrasomics can also analyze the molecular and biological characteristics of tumors, providing a scientific basis for targeted treatment programs. Regarding follow-up information, ultrasomics analysis on images before and after treatment can predict treatment effects and patient survival, thus assisting in the development of individualized and precise treatment plans. In this review, we briefly introduce the applications of ultrasomics in the breast, liver, and thyroid (Table 1).

**TABLE 1:** Summary of ultrasomics studies in oncology.

Stydies	Study design	Cancer	No. of patients	Modality	Features	Feature classifier	Type of features	Statistical analysis	Endpoint	Result
Zhao et al. (37)	Retrospective Single center	Liver	177	BMUS/SWE/SWV	2560	SRT/SVM	GM/GEM/GEVM	Mann–Whitney *U* test	prognosis and diagnosis	AUC: 0.94 (benign/malignant) AUC: 0.97 (malignant subtyping) AUC: 0.97 (PD-1 prediction) AUC: 0.94 (Ki-67 prediction) AUC: 0.98 (MVI prediction)
Zhou et al. (20)	Retrospective Single center	Breast	205	SWE	4224	CNN	—	—	Diagnosis	Accuracy: 95.8% Sensitivity: 96.2% Specificity: 95.7%
Li et al. (21)	Retrospective Single center	Breast	178	BMUS/SWE/CEUS	1226	SVM	Intensity/Texture/Contourlet/Shape/Perfusion	Holdout test	Diagnosis	Accuracy: 84.12% Sensitivity: 92.86% Specificity:78.80% AUC: 0.919
Luo et al. (26)	Retrospective Single center	Breast	315	BMUS	1044	LASSO	Histogram/Texture/RLM/Form factor	Multivariate regression analysis	Diagnosis	AUC: 0.928
Lee et al. (30)	Retrospective Single center	Breast	901	BMUS	730	LASSO	Intensity/Texture/Wavelet	—	Diagnosis	AUC: 0.782
Zhang et al. (14)	Retrospective Single center	Breast	117	Sonoelastography	364	clusters derived	Shape/intensity/GLCM/contourlet	Clusters derived/SVM	Diagnosis	AUC: 0.97 Accuracy: 88.0% Sensitivity: 85.7% Specificity: 89.3%
Qiu et al. (31)	Retrospective Single center	Lymph node	256	BMUS	843	LASSO and ridge regression	Shape/firstorder GLCM/gray-level size zone matrix/gray-level distance zone matrix/neighborhood gray-tone difference matrix/gray-level run length matrix	Elastic net logistic regression	Diagnosis	AUC: 0.816
Li et al. (33)	Prospective Single center	Liver	144	BMUS/CEMF	472	Spearman’s correlation coefficient	Conventional radiomics/ORF/CEMF features	—	Diagnosis	Mean AUC: 0.78–0.85 (the multiparametric ultrasomics model)
Wang et al. (34)	Prospective Multicentre	Liver	654	SWE	—	CNN	—	Student’s *t* test/Mann–Whitney *U* test	Prognosis	AUC: 0.97 (F4) AUC: 0.98 (F3) AUC: 0.85 (F2)
Hu et al. (38)	Retrospective Multicentre	Liver	482	CEUS	1044	LASSO	—	LASSO	Prognosis	AUC: 0.731 *p* = 0.015
Liang et al. (39)	Retrospective Multicentre	Thyroid	137	BMUS	1044	LASSO	—	Univariate logistic regression	Diagnosis	AUC: 0.921 (training cohort) AUC: 0.931 (validation cohort)
Liu et al. (40)	Retrospective Single center	Lymph node	1216	BMUS	614	combined feature selection strategy	Echo/posterior acoustic/calcification	—	Prognosis	AUC: 0.782
Park et al. (41)	Retrospective Single center	Thyroid	768	BMUS	730	LASSO	—	LASSO/Cox regression	Prognosis	C-index: 0.777; 95%[CI]: 0.735, 0.829
Liu et al. (42)	Retrospective Single center	Lymph node	75	BMUS/SE-US	684	SVM	—	Delong’s test	Prognosis	AUC: 0.90 Accuracy: 0.85 Sensitivity: 0.77 Specificity: 0.88

*The design of the studies, category of tumors, number of patients, number of features, type of features, mode build method, endpoint, diagnostic modality, and results of the studies were considered. The name of the first author and the reference number are indicated in the first column. BMUS, B-mode ultrasound; SWE, shear wave elastography; SWV, shear wave viscosity; CEUS, contrast-enhanced ultrasound; SE-US, strain ultrasound elastography; GM, the gray-scale modality; GEM, the gray-scale and elastography modality; GEVM, gray-scale, elastography and viscosity modality; SVM, support vector machine; LASSO, least absolute shrinkage and selection operator; RLM, gray level run-length matrix; CEMF, contrast-enhanced micro-flow; ORF, original radiofrequency; CEMF, contrast-enhanced micro-flow; and SRT, sparse representation theory.*

### Breast

Breast cancer is a major health problem in women. The early detection and identification of breast tumors is of great importance for improving quality of life. There are an increasing number of reports on the application of ultrasomics in breast diseases.

#### Screening, Diagnosis, Classification, and Staging

In traditional ultrasound diagnosis, images are purely used as pictures for human visual interpretation. This process relies heavily on the subjective scoring of images and the limited sensitivity of the naked eye. It can only extract completely macroscopic disease features and misses several pieces of important microbiological information (19). Ultrasomics extracts high-throughput information and performs quantitative analysis with a CAD, which can objectively describe and explain the features of tumors. At present, studies have combined conventional 2D ultrasound images, shear-wave elastography (SWE) images, strain elastography images, and contrast-enhanced ultrasound (CEUS) images with radiomics to detect and identify breast tumors (14, 20–22). These studies extracted high-throughput features to quantify tumor shape, hardness, and hardness heterogeneity to identify breast malignancies and benign tumors. Moreover, these studies found that quantitative ultrasound features were significantly associated with hormone receptor status, molecular subtype and histologic grade in breast invasive ductal carcinoma (IDC). Ultrasomics also makes it possible to evaluate biological parameters by non-invasive means (23–25). Moreover, in a study by Luo et al. 19 features selected by Least absolute shrinkage and selection operator (LASSO) were used to score the degree of malignancy of Breast Imaging Reporting and Data System (BI-RADS) 4 and 5 breast masses, and they obtained AUC values of 0.921 and 0.931 in the training and validation groups, respectively (26). This study showed the outstanding discriminative ability of ultrasomics in grading the possibility of malignancy. Ultrasomics reflects tissue structure and morphological features by quantitatively analyzing the gray value of medical images and then extracting the quantitative features with computer algorithms. This approach can effectively avoid the subjective description of radiologists and the large variability between observers (20, 27). Ultrasomics clarifies the correlation between the malignant potential of masses and image features and shows good prospects for tumor diagnosis.

#### Individualized Treatment and Survival Prediction

The core task of precision medicine is to identify patient phenotypes (disease, treatment response, adverse side effects, and survival prediction) to find individualized treatment options. Cancer cells exhibit a high degree of heterogeneity, even in different regions of the same tumor, different metastatic sites in the same patient, or the same type of tumor among different patients. This high degree of genetic variation explains the failure of targeted therapies and allows the emergence and proliferation of resistant clones (28). In this case, techniques for quantifying intra- and intertumor heterogeneity are critical because they may guide adaptive treatment (29). Lee et al. performed ultrasomics scoring on 901 lesions and ultimately obtained a model for distinguishing triple-negative breast cancer (TNBC) from breast fibroadenomas (30). Texture features were extracted using the GLCM and GLRLM in this study, they found that both tumor grade and receptor status had an impact on ultrasound performance. Tumors with ER^+^, her 2^–^ are associated with irregular shapes, unbounded edges, or complex echo patterns, and rear shadowing. This may be caused by the relatively slow proliferation rate of cells, the long-term interaction between a tumor and host, and hyperplasia of the fibrous tissue around the lesion, which results in uneven borders, burrs or leaves. Interstitial response and connective tissue hyperplasia cause different acoustic impedance differences, acoustic reflections, and echo attenuation behind the mass. However, TNBC tends to have oval or round shapes and circumscribed margins, reflecting a rapidly proliferating tumor prior to significant stromal reaction. It is also more likely to present with posterior acoustic enhancement since highly cellular circumscribed carcinomas tend to have enhanced through-transmission. This benign-looking might decrease the diagnostic efficacy of ultrasound and delay treatment. Radiomics based on texture analysis shows excellent diagnostic performance in the differential diagnosis of fibroadenoma and TNBC where it is indiscernible with the naked eye. Theoretically, ultrasound images may contain hidden information that can be difficult for radiologists to mine (29). Ultrasomics can find heterogeneities within a region from indistinguishable imaging data.

Sentinel lymph nodes are an important factor for the prognosis of breast cancer patients. The precise and non-invasive prediction of axillary lymph nodes before surgery is of great significance for staging, treatment and prognosis. Qiu X et al. combined ultrasomics with features of axillary lymph nodes on B-mode ultrasound images and found that a radiomics model with LASSO and ridge regression methods was able to predict axillary lymph node metastasis by using ultrasound features of primary breast tumors (31). This strategy might be an effective alternative to early screening for lymph node metastasis in clinically lymph node–negative breast cancer. It also showed the great potential to serve as an important decision support tool in clinical practice. It is expected to reduce the axillary lymph node dissection and sentinel lymph node biopsy and the corresponding postoperative complications accordingly.

### Liver

#### Screening, Diagnosis, Classification, and Staging

Hepatitis B virus (HBV) infection is a serious problem around the world. Liver fibrosis, cirrhosis, and liver cancer are progressive diseases of chronic hepatitis B (CHB). An accurate assessment of liver status is essential for the prognosis, monitoring and management of CHB patients. D Souza et al. studied the B-mode ultrasound features of the liver in a rat model to assess liver fibrosis (32). The computer algorithm extracted quantitative parameters representing brightness (echo intensity and liver and kidney index) and variance (heterogeneity) to study the anisotropy of the liver. The echo intensity of DEN rats increased from 37.1 ± 7.8 to 53.5 ± 5.7∼57.5 ± 6.1, compared with an average of 34.5 ± 4.5 in the control group. Histological analysis revealed that fibrosis fractionation with METAVIR scores F2-F4 and specifically F0-F1 in DEN rats increased the imaging parameters. Wang et al. and Li et al. applied this technique in clinical practice. Li et al. acquired ultrasound radio frequency signals and dynamic perfusion information to construct an ultrasomics model, and they derived an optimal algorithm for assessing liver fibrosis in a small sample (33). Wang et al. suggested that deep learning radiomics of elastography (DLRE) could be successfully used to assess the liver fibrosis stage of patients with CHB and was comparable to the current grading criteria for cirrhosis and advanced fibrosis. The diagnostic accuracy of the model was higher than that of 2D-SWE for overcoming the influence of inflammation on cirrhosis assessments (34). Therefore, ultrasomics is a potential breakthrough in image diagnosis.

#### Individualized Treatment and Survival Prediction

Ultrasomics applies the identification, analysis, and integration of ultrasound images to reach a better solution for patients. The main factors in the recurrence of liver cancer are microvascular infiltration (MVI) and Ki-67 (35). MVI is a common predictor of the prognosis for patients with liver cancer. MVI is highly correlated with early recurrence and greatly influences treatment (liver resection or orthotopic liver transplant) (36). Yao et al. classified images by transforming them into high-throughput features, analyzed multiple parameters in the treatment area and used sparse representation theory (SRT), and support vector machine (SVM) methods to mine rich texture information. The authors found that malignant tumors had more complex textures and structural information than benign tumors. Their results indicated that predicting MVI (AUC = 0.98), Ki-67 (AUC = 0.94), and PD-1 (AUC = 0.97) with a non-invasive method based on radiomics is feasible (37). This finding showed that ultrasomics could improve the diagnostic efficiency of ultrasound and made it possible to diagnose FLL before operation. Additionally, ultrasomics can explain the biological behavior of tumors and improve the diagnostic efficacy and patient prognosis (38).

### Thyroid

#### Screening, Diagnosis, Classification, and Staging

Thyroid disease has received widespread attention due to its high incidence. The Thyroid Imaging-Reporting and Data System (TI-RADS) is widely used to describe thyroid lesions and is unavoidably subjective. In a study, Liang et al. developed an ultrasomics model to diagnose malignant thyroid nodules. They performed LASSO to select features and found that ultrasomics could outperform ACR TI-RADS scoring, at least when performed by junior radiologists (39). In addition, the application of texture analysis and machine learning in thyroid nodule imaging can describe thyroid nodules better and more objectively. Liu et al. obtained predictive models though a SVM classifier from more than 50 traits of thyroid tumors, such as the volume, echo, margin, boundaries, posterior acoustic pattern, and calcification features. They obtained satisfactory results in predicting which thyroid nodules would develop lymph node metastasis (40). Lymph node metastasis is more likely to occur in patients with complex echoes in ultrasound images, uniform posterior regions, large calcifications or multiple calcifications (41). Clinically, the lymph nodes suspected malignant are re-examined by CT, fine needle aspiration cytology, or lymph node dissection (LND). LND has the risk of hyperparathyroidism and nerve injury. In addition, whether LND can improve the survival rate of PTC patients is still controversial, so full consideration must be given before it is used in patients. The radiomics evaluation has potential to predict LN status non-invasively based on preoperative ultrasound thyroid images. It made up for the shortcomings of traditional diagnosis. The lymph node status prediction model has the potential to promote early medical management for thyroid cancer patients and reduce overdiagnosis.

#### Individualized Treatment and Survival Prediction

Recurrence and metastasis are the key points during the treatment of cancer and are closely related to the survival time of patients. Long-term follow-up assessments are indispensable after tumor treatment. The most important part of this process is to determine whether there are any recurrences or residual lesions. Liu et al. combined the features extracted from B-mode ultrasound and strain ultrasound elastography (SE-US), and multimodal feature sets were obtained through image segmentation, quantitative feature extraction, feature selection and classification. This study used the sparse representation coefficient-based feature selection method with 10 bootstraps to reduce the dimensionality of the feature sets. A SVM with leave-one-out cross-validation was used to build the model to estimate LN status. The model had the best ability to diagnose lymph node metastases (42). Furthermore, ultrasomics could not only characterize the properties of thyroid nodules but also assess the disease-free survival of thyroid nodule patients. This is the first application of ultrasomics to predict the prognosis of thyroid cancer. The authors used relapsed or persistent disease-free survival as the study endpoint rather than mortality, demonstrating the great potential of ultrasomics (41).

## Challenges

First and foremost, the greatest problem with ultrasomics is the quality and quantity of the original data. A successful ultrasomics model needs a sufficient quantity of data to develop an effective knowledge system to support data integration, processing, and analysis, which is critical for research to be optimal. Currently, ultrasomics usually use a smaller population to extract more features, which may lead to overfitting and overoptimistic results. There are numerous methods for extracting useful biomarkers from separate or combined layers of ultrasomics and clinical data, but the results are still unsatisfactory. Second, ultrasound examinations are less reproducible than other imaging methods. Additionally, the device and experience of the radiologist have a great impact on the reliability of the diagnosis (43). Therefore, the inclusion and exclusion criteria for ultrasomics should be rigorously developed. Recently, the image biomarker standardization initiative (IBSI) was proposed to improve the reproducibility of high-throughput imaging analysis, which is a valuable step in improving radiological research. In addition, a radiomics quality score (RQS) was proposed to help evaluate radiomics studies (44). Moreover, most of the published ultrasomics studies are from a single center, with different patient numbers, different ultrasound equipment, and different study design methods. The differences in each step of the study design pose greater challenges to the repeatability of the study.

## Conclusion and Perspectives

Although many problems still need to be solved, the potential of ultrasomics is beyond doubt, and the field is evolving rapidly. The development of ultrasomics has occurred over only a few decades, and some impressive results have been achieved. This approach fills the gap in the clinical use of information and extracts and analyzes higher-dimensional and quantitative data to more accurately and more specifically describe and characterize tumors. The use of ultrasomics to improve disease diagnosis and care for patients shows great potential (45). In the future, we hope that ultrasomics will provide a more personalized, higher-quality, and more cost-effective care platform for patients. The advantages of ultrasomics, including its speed, low cost, reproducibility, and non-invasiveness, may make it a valuable clinical decision-making tool.

## Author Contributions

X-WC, BH, and CD established the design and conception of the manuscript. RY, MJ, W-ZL, FJ, and JL explored the literature data. RY provided the first draft of the manuscript, which was discussed and revised critically for intellectual content by RY, MJ, W-ZL, FJ, JL, BH, X-WC, and CD. All authors discussed the statement and conclusions and approved the final version to be published.

## Conflict of Interest

The authors declare that the research was conducted in the absence of any commercial or financial relationships that could be construed as a potential conflict of interest.

## References

[1] Gillies RJ Kinahan PE Hricak H. Radiomics: images are more than pictures, they are data. *Radiology.* (2016) 278:563–77. 10.1148/radiol.2015151169 26579733 PMC4734157

[2] Avanzo M Stancanello J El Naqa I. Beyond imaging: the promise of radiomics. *Phys Med.* (2017) 38:122–39. 10.1016/j.ejmp.2017.05.071 28595812

[3] Li Q Ye ZX. Radiomics: the process and applications in tumor research. *Chin J Oncol.* (2018) 40:801–4. 10.3760/cma.j.issn.0253-3766.2018.11.001 30481928

[4] Liu Z Wang S Dong D Wei J Fang C Zhou X The applications of radiomics in precision diagnosis and treatment of oncology: opportunities and challenges. *Theranostics.* (2019) 9:1303–22. 10.7150/thno.30309 30867832 PMC6401507

[5] Pinker K Chin J Melsaether AN Morris EA Moy L. Precision medicine and radiogenomics in breast cancer: new approaches toward diagnosis and treatment. *Radiology.* (2018) 287:732–47. 29782246 10.1148/radiol.2018172171

[6] Kumar V Gu Y Basu S Berglund A Eschrich SA Schabath MB Radiomics: the process and the challenges. *Magn Reson Imaging.* (2012) 30:1234–48. 10.1016/j.mri.2012.06.010 22898692 PMC3563280

[7] Holzinger A Haibe-Kains B Jurisica I. Why imaging data alone is not enough: AI-based integration of imaging, omics, and clinical data. *Eur J Nucl Med Mol Imaging.* (2019) 46:2722–30. 31203421 10.1007/s00259-019-04382-9

[8] Keek SA Leijenaar RT Jochems A Woodruff HC. A review on radiomics and the future of theranostics for patient selection in precision medicine. *Br J Radiol.* (2018) 91:20170926. 10.1259/bjr.20170926 PMC6475933 29947266

[9] Sollini M Antunovic L Chiti A Kirienko M. Towards clinical application of image mining: a systematic review on artificial intelligence and radiomics. *Eur J Nucl Med Mol Imaging.* (2019) 46:2656–72. 10.1007/s00259-019-04372-x 31214791 PMC6879445

[10] Tran WT Jerzak K Lu FI Klein J Tabbarah S Lagree A Personalized breast cancer treatments using artificial intelligence in radiomics and pathomics. *J Med Imaging Radiat Sci.* (2019) 50:S32–41. 10.1016/j.jmir.2019.07.010 31447230

[11] Hatt M Le Rest CC Tixier F Badic B Schick U Visvikis D. Radiomics: data are also images. *J Nucl Med.* (2019) 60:38S–44S. 10.2967/jnumed.118.220582 31481588

[12] Ma M Feng Z Peng T Yan H Rong P Jumbe MM. Radiomics and its advances in hepatocellular carcinoma. *Zhong Nan Da Xue Xue Bao Yi Xue Ban.* (2019) 44:225–32. 10.11817/j.issn.1672-7347.2019.03.001 30971513

[13] Elter M Horsch A. CADx of mammographic masses and clustered microcalcifications: a review. *Med Phys.* (2009) 36:2052–68. 19610294 10.1118/1.3121511

[14] Zhang Q Xiao Y Suo J Shi J Yu J Guo Y Sonoelastomics for breast tumor classification: a radiomics approach with clustering-based feature selection on sonoelastography. *Ultrasound Med Biol.* (2017) 43:1058–69. 10.1016/j.ultrasmedbio.2016.12.016 28233619

[15] Korn RL Rahmanuddin S Borazanci E. Use of precision imaging in the evaluation of pancreas cancer. *Cancer Treat Res.* (2019) 178:209–36. 10.1007/978-3-030-16391-4_8 31209847

[16] Drabycz S Stockwell RG Mitchell JR. Image texture characterization using the discrete orthonormal S-transform. *J Digit Imaging.* (2009) 22:696–708. 10.1007/s10278-008-9138-8 18677534 PMC2782119

[17] Khan AM El-Daly H Simmons E Rajpoot NM. HyMaP: a hybrid magnitude-phase approach to unsupervised segmentation of tumor areas in breast cancer histology images. *J Pathol Inform.* (2013) 4:S1. 10.4103/2153-3539.109802 23766931 PMC3678741

[18] Lambin P Leijenaar RTH Deist TM Peerlings J de Jong EEC van Timmeren J Radiomics: the bridge between medical imaging and personalized medicine. *Nat Rev Clin Oncol.* (2017) 14:749–62. 10.1038/nrclinonc.2017.141 28975929

[19] Valdora F Houssami N Rossi F Calabrese M Tagliafico AS. Rapid review: radiomics and breast cancer. *Breast Cancer Res Treat.* (2018) 169:217–29. 10.1007/s10549-018-4675-4 29396665

[20] Zhou Y Xu J Liu Q Li C Liu Z Wang MA. Radiomics approach with CNN for shear-wave elastography breast tumor classification. *IEEE Trans Biomed Eng.* (2018) 65:1935–42. 29993469 10.1109/TBME.2018.2844188

[21] Li Y Liu Y Zhang M Zhang G Wang Z Luo J. Radiomics with attribute bagging for breast tumor classification using multimodal ultrasound images. *J Ultrasound Med.* (2020) 39:361–71. 10.1002/jum.15115 31432552

[22] Sollini M Cozzi L Chiti A Kirienko M. Texture analysis and machine learning to characterize suspected thyroid nodules and differentiated thyroid cancer: where do we stand? *Eur J Radiol.* (2018) 99:1–8. 10.1016/j.ejrad.2017.12.004 29362138

[23] Bodalal Z Trebeschi S Beets-Tan R. Radiomics: a critical step towards integrated healthcare. *Insights Imaging.* (2018) 9:911–4. 10.1007/s13244-018-0669-3 30421396 PMC6269340

[24] Ko ES Lee BH Kim HA Noh WC Kim MS Lee SA. Triple-negative breast cancer: correlation between imaging and pathological findings. *Eur Radiol.* (2010) 20:1111–7. 19898850 10.1007/s00330-009-1656-3

[25] Çelebi F Pilancı KN Ordu Ç Ağacayak F Alço G İlgün S The role of ultrasonographic findings to predict molecular subtype, histologic grade, and hormone receptor status of breast cancer. *Diagn Interv Radiol.* (2015) 21:448–53. 10.5152/dir.2015.14515 26359880 PMC4622390

[26] Luo WQ Huang QX Huang XW Hu HT Zeng FQ Wang W Predicting breast cancer in breast imaging reporting and data system (BI-RADS) ultrasound category 4 or 5 lesions: a nomogram combining radiomics and BI-RADS. *Sci Rep.* (2019) 9:11921. 10.1038/s41598-019-48488-4 PMC6695380 31417138

[27] Gibbs P Onishi N Sadinski M Gallagher KM Hughes M Martinez DF Characterization of sub-1 cm breast lesions using radiomics analysis. *J Magn Reson Imaging.* (2019) 50:1468–77. 10.1002/jmri.26732 30916835 PMC8500553

[28] Rossi SH Prezzi D Kelly-Morland C Goh V. Imaging for the diagnosis and response assessment of renal tumours. *World J Urol.* (2018) 36:1927–42. 10.1007/s00345-018-2342-3 29948048 PMC6280818

[29] Nougaret S Tibermacine H Tardieu M Sala E. Radiomics: an introductory guide to what it may foretell. *Curr Oncol Rep.* (2019) 21:70. 10.1007/s11912-019-0815-1 31240403

[30] Lee SE Han K Kwak JY Lee E Kim EK. Radiomics of US texture features in differential diagnosis between triple-negative breast cancer and fibroadenoma. *Sci Rep.* (2018) 8:13546. 10.1038/s41598-018-31906-4 PMC6131410 30202040

[31] Qiu X Jiang Y Zhao Q Yan C Huang M Jiang T. Could ultrasound-based radiomics noninvasively predict axillary lymph node metastasis in breast cancer? *J Ultrasound Med.* (2020). 10.1002/jum.15294 [Epub ahead of print]. 32329142 PMC7540260

[32] D‘Souza JC Sultan LR Hunt SJ Schultz SM Brice AK Wood AKW B-mode ultrasound for the assessment of hepatic fibrosis: a quantitative multiparametric analysis for a radiomics approach. *Sci Rep.* (2019) 9:8708. 10.1038/s41598-019-45043-z 31213661 PMC6581954

[33] Li W Huang Y Zhuang BW Liu GJ Hu HT Li X Multiparametric ultrasomics of significant liver fibrosis: a machine learning-based analysis. *Eur Radiol.* (2019) 29:1496–506. 30178143 10.1007/s00330-018-5680-z PMC6510867

[34] Wang K Lu X Zhou H Gao Y Zheng J Tong M Deep learning Radiomics of shear wave elastography significantly improved diagnostic performance for assessing liver fibrosis in chronic hepatitis B: a prospective multicentre study. *Gut.* (2019) 68:729–41. 10.1136/gutjnl-2018-316204 29730602 PMC6580779

[35] Ni M Zhou X Lv Q Li Z Gao Y Tan Y Radiomics models for diagnosing microvascular invasion in hepatocellular carcinoma: which model is the best model? *Cancer Imaging.* (2019) 19:60. 10.1186/s40644-019-0249-x PMC6712704 31455432

[36] Portolani N Coniglio A Ghidoni S Giovanelli M Benetti A Tiberio GA Early and late recurrence after liver resection for hepatocellular carcinoma: prognostic and therapeutic implications. *Ann Surg.* (2006) 243:229–35. 10.1097/01.sla.0000197706.21803.a1 16432356 PMC1448919

[37] Yao Z Dong Y Wu G Zhang Q Yang D Yu JH Preoperative diagnosis and prediction of hepatocellular carcinoma: radiomics analysis based on multi-modal ultrasound images. *BMC Cancer.* (2018) 18:1089. 10.1186/s12885-018-5003-4 30419849 PMC6233500

[38] Hu HT Wang Z Huang XW Chen SL Zheng X Ruan SM Ultrasound-based radiomics score: a potential biomarker for the prediction of microvascular invasion in hepatocellular carcinoma. *Eur Radiol.* (2019) 29:2890–901. 10.1007/s00330-018-5797-0 30421015

[39] Liang J Huang X Hu H Liu Y Zhou Q Cao Q Predicting malignancy in thyroid nodules: radiomics score versus 2017 american college of radiology thyroid imaging, reporting and data system. *Thyroid.* (2018) 28:1024–33. 10.1089/thy.2017.0525 29897018

[40] Liu T Zhou S Yu J Guo Y Wang Y Zhou J Prediction of lymph node metastasis in patients with papillary thyroid carcinoma: a radiomics method based on preoperative ultrasound images. *Technol Cancer Res Treat.* (2019) 18:1533033819831713. 10.1177/1533033819831713 30890092 PMC6429647

[41] Park VY Han K Lee E Kim EK Moon HJ Yoon JH Association between radiomics signature and disease-free survival in conventional papillary thyroid carcinoma. *Sci Rep.* (2019) 9:4501. 10.1038/s41598-018-37748-4 30872763 PMC6418281

[42] Liu T Ge X Yu J Guo Y Wang Y Wang W Comparison of the application of B-mode and strain elastography ultrasound in the estimation of lymph node metastasis of papillary thyroid carcinoma based on a radiomics approach. *Int J Comput Assist Radiol Surg.* (2018) 13:1617–27. 29931410 10.1007/s11548-018-1796-5

[43] Rix A Lederle W Theek B Lammers T Moonen C Schmitz G Advanced ultrasound technologies for diagnosis and therapy. *J Nucl Med.* (2018) 59:740–6. 29496981 10.2967/jnumed.117.200030

[44] Radiomics (2020). Available online at: http://www.radiomics.world/ (accessed June 28, 2020).

[45] Forghani R Savadjiev P Chatterjee A Muthukrishnan N Reinhold C Forghani B. Radiomics and artificial intelligence for biomarker and prediction model development in oncology. *Comput Struct Biotechnol J.* (2019) 17:995–1008. 10.1016/j.csbj.2019.07.001 31388413 PMC6667772

